# Blended intensive programme’s implementation in dental education: post-pandemic evolution of learning

**DOI:** 10.1186/s12909-024-05301-9

**Published:** 2024-03-29

**Authors:** Irena Duś-Ilnicka, Anna Paradowska-Stolarz, Marta Mazur, Małgorzata Radwan-Oczko, Andrea Perra, Vanessa Salete de Paula, Laura Sterian Ward, Nicola Alberto Valente, Elena Firkova, Teodora Karteva, Lucia Miralles Jorda, Pedro de Sousa Gomes, Marzena Dominiak

**Affiliations:** 1 Department of Oral Pathology, Wrocław Medical University, ul. Krakowska 26, 50-425 Wrocław, Poland; 2 Department of Dentofacial Anomalies, Department of Orthodontics and Dentofacial Orthopedics, Wroclaw Medical University, Krakowska 26, 52-425 Wrocław, Poland; 3 Department of Oral and Maxillofacial Sciences, Sapienza University of Rome, 00161 Rome, Italy; 4 Sezione di Patologia, Dipartimento di Scienze biomediche, Università degli Studi di Cagliari, Cittadella Universitaria, Monserrato, Italy; 5 Molecular Virology and Parasitology Laboratory, IOC/ Oswaldo Cruz Foundation, Rio de Janeiro, Brazil; 6 Laboratory of Cancer Molecular Genetics, School of Medical Sciences, State University of Campinas (Unicamp), Campinas, Brazil; 7 Division of Periodontology, School of Dental Medicine, Department of Surgical Sciences, Faculty of Medicine and Surgery, University of Cagliari, Cagliari, Italy; 8 Department of Periodontology and Oral Diseases, Faculty of Dental Medicine, Medical University of Plovdiv, Plovdiv, Bulgaria; 9 Department of Operative Dentistry and Endodontics Faculty of Dental Medicine, Medical University Plovdiv, Plovdiv, Bulgaria; 10 Dentistry Department, Medical and Health Sciences Faculty, Catholic University of Valencia, Calle Quevedo, 2, 46001 Valencia, Spain; 11 Laboratory for Bone Metabolism and Regeneration, Faculty of Dental Medicine, University of Porto, 4200-393 Porto, Portugal; 12 Oral Surgery Department, Wroclaw Medical University, Krakowska 26, 50-425 Wroclaw, Poland

**Keywords:** Hybrid programmes, Dentistry teaching, Interprofessional, International

## Abstract

Blended Intensive Programmes (BIP’s) represent a valuable tool for gathering knowledge and summarising the latest trends in medicine and dentistry. Blended education has been found, even before the COVID-19 pandemic, to increase the level of education and stimulate effective learning for postgraduate healthcare professionals. Interprofessional education is critical for preparing students to enter the health workforce, where teamwork and collaboration are important competencies. This article outlines the key points of the Blended Intensive Programme’s implementation in dental education organised by Wroclaw Medical University in Poland. BIP involved professors from 12 universities or research institutions from Europe and South America and 28 participants from 8 countries. The course was taught remotely and in person. In addition, it included a visit to the university and practical classes with artificial simulation and practice in dentistry. A structured questionnaire enabled measuring the evaluation of students’ perception of the COVID-19 education before and after the pandemic. The European Region Action Scheme for the Mobility of University Students (ERASMUS) was fundamental to carrying out the BIP with the participation of several countries, allowing the exchange of knowledge, assessing the impact of the pandemic on dental universities, and strengthening international collaborations and the future project of research, education and clinical assistance. We conclude that hybrid teaching programmes broaden the learning spectrum in dental studies by allowing transnational and interdisciplinary approaches that make students aware of the importance of their work within the framework of the general health approach, as this differs from country to country.

**Supplementary Information:**

The online version contains supplementary material available at 10.1186/s12909-024-05301-9.

## Introduction

The landscape of education in clinical disciplines, particularly dentistry, underwent unprecedented challenges during the COVID-19 pandemic. The disruption was not solely confined to the unavailability of in-person lectures, a significant setback as it deprived students of interpersonal exchanges and direct interaction with educators. Beyond this, a profound impact was felt in the realm of regular clinical training, a cornerstone of practical education in dentistry [1]. The inability to conduct routine clinical training sessions posed a multifaceted challenge, raising concerns about the comprehensive development of students in a discipline where hands-on experience is paramount. Although medical (including dental) studies are challenging for students and require a lot of practice, the pandemic developed e-learning as a more popular and available method [2]. COVID-19 pandemics showed, that implementation of some of the educational workload by the introduction of the online learning, represented in the blended version, can be benefitial to the learning process [1, 3]. In this context, we explore how these challenges served as the catalyst for the inception of innovative approaches, such as the Blended Intensive Programme (BIP), aiming to navigate the complexities imposed by the pandemic and redefine the landscape of dental education.

Apart from previously stated, dental education stood out as one of the earliest and most significantly impacted domains of higher education to be affected by the COVID-19 measures aimed at diminishing the risk of microbiological contamination. Common reasoning was provided, as this clinical profession has been associated with a major risk of infection during the COVID-19 pandemic [4–6] because of the risk of SARS-CoV-2 transmission from the respiratory tract of patients due to work in the oral cavity, with the use of equipment generating bioaerosol [7]. For this reason, special precautions were implemented during the pandemic. At the beginning of 2020, the personal protective equipment scarcity due to delays in shipment inadvertently amplified concerns about potential infections among dentists, and some dental clinics were closed for some time [6, 8], compounding the challenges. Additionally, some teaching hospitals, often fostering close student-patient clinical interactions in the open-plan environment [9, 10], introduced protocols that only urgent procedures should be performed (such as those associated with the risk of loss of a tooth or pain reported by the patient) [11, 12]. Unsurprisingly, this has affected the practical training of students. Also, the possibility of performing the international exchanges within the 2 first years of the pandemic was scarce to non-existent, what has influenced negatively the internationalisation of the intstitutions.

In response, the education paradigm underwent a significant shift, with the pandemic ushering in an era of comprehensive online learning for future dentists. This was performed entirely online, using the remote learning possibilities, or in some parts, through a blended version in a hybrid format. This trend, the rapid change in education practice, extended beyond dental education into other fields of medical training [3, 13]. The disruptions brought by the pandemic have galvanised the education sector to adapt, transform and innovate, introducing novel pedagogical approaches to ensure the continuity of learning despite unparalleled obstacles.

According to the European Commission (EC) perspective, blended mobility, described here in reference to its use in the Blended Intensive Programmes and recognised under this specific name in European higher education, is a combination of physical mobility with a virtual component facilitating a collaborative online learning exchange and teamwork. This project work frame provided by EC is a part of the European Region Action Scheme for the Mobility of University Students (ERASMUS+) domain, where the implementation of the physical activity comprises a minimum of 5 days of in-person meetings, while the online implementation of virtual components is not given a specific timeframe. During a single BIP, the number of participants ranges from 15 to 60 learners, understood as teachers and/or students depending on the type of the program selected for the BIP. For the students, BIP provides a minimum of 3 credits under the European Credit Transfer and Accumulation System (ECTS) for the activity and a maximum of 6 ECTS, depending on the declared schedule. To make the programme eligible, learners can participate in the BIP using ERASMUS + funds or their personal funds [14]. BIPs may include challenge-based learning with the special implementation of transnational and interdisciplinary teams working together in order to address societal challenges faced by specific regions, cities or businesses. The one proposed action is to implement some of the prerequisites of growth provided by the UN Sustainable Development Goals [15]. Additionally, as outlined by the EU, BIPs must provide added value in comparison to the existing courses or training offered by participating institutions [14]. While participation of learners from outside the EU region is possible, these participants are not considered for meeting the minimal number of required participants for the eligibility criteria to be met.

As described above, the BIP teaching concepts provide a suitable solution for the current need of the dental medicine teaching/learning process for switching to the remote and blended versions with the perfect use of its novelty and relevance factors. Here described Blended Intensive Programme (BIP) “DentalOmics: Transnational agreement for interdisciplinary dentistry”, organised collaboratively by all partners, embraced three overarching goals. Firstly, it sought to gather insights from both students and teachers about the practice during the pandemic and the use of telemedical equipment and artificial intelligence (AI) in dental studies. In the ever-evolving landscape of dentistry, the integration of AI is emerging as a promising frontier, offering both potential advantages and presenting unique challenges. Secondly, the focus was put on the implementation and analysis of the laboratory results from different fields, so the general medicine professionals were invited to share and exchange ideas about data analysis (including mega-data). Thirdly, general discussion was provided during our BIP between dental and medical professionals, with a common goal– recognising that oral health reflects general health and the way of teaching using tutoring skill sets in one-by-one settings. The latter two goals were implemented to fulfil the goal of presenting an interdisciplinary collaboration among dentists and other medical professions. It was our intention to create an environment that promotes the exchange of insights and expertise across diverse medical disciplines.

Accordingly, with this manuscript, we aimed to shed light on the technical intricacies, programme implementation and teaching methodologies of an innovative BIP in dental medicine rather than delving into specific descriptions of the seminars and dental topics covered during the programme. Our hope is that by sharing these broader insights, we contribute to the ongoing dialogue about the vital intersection between dentistry and various medical disciplines.

## Methodology of the programme preparation

### Ethical commite agreement for the use of personal data

This research on didactics processes in internationalisation has received the waiver of the requirement for the signed ethical agreement from the participants of this project. This project has received the positive opinion of the Wroclaw Medical University Bioethical Committee, number KB 272/2023N.

### Recruitment of the participants

**Fig. 1:**
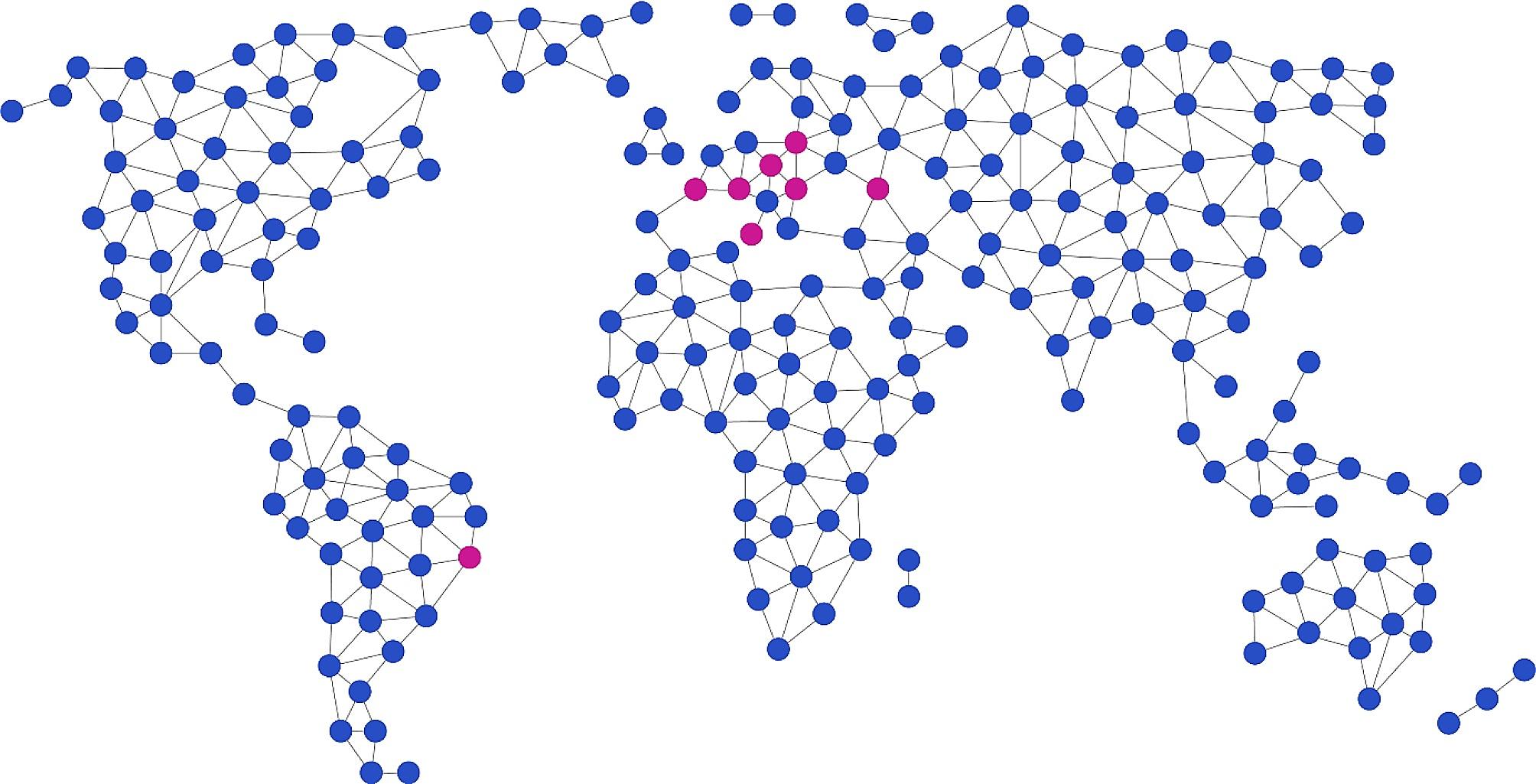
Geographical description of the participants of BIP “DentalOmics”

Participants from the EU region and participants included in the KA171 + programme are presented in Table 1; Fig. 1. Application to the participation in the “in person” component was organised by the partner HEIs; however, the form/questionnaire for the participants and description of the application was provided to the HEIs by the leader. HEIs provided the time of 14 days to 1 month for the participants to be chosen. One university from the EU region (Faculty of Dentistry of the University of Turin, Italy) participated only in the online component by providing seminars by two dental professors; one of them was the ERASMUS + Coordinator of the Faculty.

### Topic selection and participants

The developed BIP was entitled: “DentalOmics”, with the subtitle: “Transnational agreement for interdisciplinary dentistry”. The first meetings about the project implementation started in November 2022 with international partners, and the BIP’s date was scheduled for 19 June to 23 June 2023 for the virtual (online components) and 26 June to 30 June 2023 for the meetings in-person at Wroclaw Medical University (leader of the project). The BIP has received funding from the European Commission following the official application for the project by the International Relations Office of Wroclaw Medical University. Topics were chosen to cover the aims of the BIP and were included in the parallel way. Each day of the virtual component had a mirror day (same topic with different techniques used) during an in-person component.

On the first day, an introductory panel included seminars, discussions and questionnaires covering the following areas:

1. How the COVID-19 pandemic has changed ERASMUS + and how did it affect dental medicine teaching?
2. What strategies and adaptations were implemented to sustain dental faculties during the first year of the pandemic? Has the situation changed in the second year?

The second day of both the online and in-person components was dedicated to the “Medicine meets dentistry panel”. In this part interdisciplinary approach to dentistry was shared by the use of advanced metagenomics approach, witht the special consideration to the discovery of potential links between general diseases and oral pathologies, dental anomalies and oral disorders.

The third day of the meetings was focused on the “Clinical dentistry panel”. On the fourth day, learners broadened their knowledge during “Laboratory meets dentistry”. Lastly, personal skills were developed on the fifth day when the “Soft skills in dentistry” were taught. This comprehensive structure ensured that learners were exposed to diverse topics, fostering interdisciplinary understanding and skill development in dental medicine.

**Table 1:** Participants in the component organised in person on the leader side at Wroclaw Medical University

		Students	Teachers	EU HEI	Total
**Leader of the BIP**	Wroclaw Medical University, Wroclaw, Poland	2	10	Yes	12
**Partners of the BIP**	University of Cagliari, Cagliari, Italy	2	2	Yes	4
	Sapienza University of Rome, Rome, Italy	0	4	Yes	4
	Medical University of Plovdiv, Bulgaria	2	2	Yes	4
	Egas Moniz School of Health & Science, Caparica, Portugal	2	0	Yes	2
**Participants**	Lithuanian University of Health Sciences, Lituania	5	0	Yes	5
	UMFST “George Emil Palade” Târgu Mureș, Romania	3	0	Yes	3
	University of Porto, Portugal	3	1	Yes	2
	Catholic University of Valencia, Spain	0	1	Yes	1
	University of Campinas, Campinas, Sao Paulo, Brazil	0	1	No	1
	Fundação Oswaldo Cruz, Rio de Janeiro, Brazil	0	1	No	1
	Caucasus International University, Tbilisi, Georgia	2	0	No	2

The BIP welcomed participants from various geographical regions to create a diverse and enriching experience. Students from Georgia’s Caucasus International University were invited to participate in the programme from outside the EU region, and teachers from Brazil were asked to enhance the meetings with their seminars and workshops. Brazilian collaborators were included under KA171 + funds received for the collaboration with the ERASMUS + coordinator of the Faculty of Dentistry in Wroclaw Medical University. Two teachers participated during the in person component, one from the University of Campinas (Campinas, São Paulo) and the second from the Fundação Oswaldo Cruz (Rio de Janeiro). ​​​​​​​​Brazilian biologists, doctors, biomedical specialists and dentists who are students of master’s or PhD students in Tropical Medicine at Fiocruz participated remotely during the online part of the programme.

### Teaching methods

The virtual component of the BIP was provided on the Teams platform designated for official use by Wroclaw Medical University and its collaborators [16]. The scheduled meetings spanned a total of 5 days. Seminars were delivered in the synchronous format, except for two provided asynchronously [17]. If the seminar was chosen to be provided in the asynchronous form, the given lecturer was still present online to address questions from the students and other participants as well as start discussions about the presented topic. Parallel to the seminars, pools on Google Spreadsheets were shared among the learners in order to provide the answers for the discussion. The use of webcams was considered optional but welcomed in the case of learners who wanted to speak during online meetings and seminar sessions.

The in-person meetings at Wroclaw Medical University were similarly divided over 5 days. These meetings encompassed the following techniques:

- Clinical job shadowing with an introductory seminar in the dental clinic divided into two parallel topics:
  1. Correct diagnosis of Oral Potentially Malignant Disorders– dentists as frontline practitioners in oral cancer diagnostics;
  2. Patients with special needs in the dental practice– a word from an orthodontist;
- Job shadowing at the Museum of Anatomy, with presentation of the preparation of basic steps of medical museum specimen processing;
- Job shadowing at the Medical Simulation Center and Virtual Reality;
- Interactive seminars of an introductory nature featuring a questionnaire assessing competencies and a summary of the acquired skills;
- Hands-on practice with the use of modern artificial intelligence techniques during the workshop;
- One-on-one tutoring meetings as needed upon request of the students during the in-person meeting.

All the components described here fulfilled the three aims described in the above methodology section.

### Cultural events

For three days of the event, the historic building of the Museum of Pharmacy in the old town of Wroclaw served as the venue. Participants had the opportunity to enhance their local experience through guided tours of the historical city centre of Wroclaw, providing them with insights into the city’s architectural landmarks. Cultural activities were thoughtfully organised for the learners, such as opportunities to learn about Polish culture, language and cuisine, sharing a multicultural dinner in the old Centennial Hall, a UNESCO World Heritage site.

## Results

### Perception of COVID-19 education by the learners (see questionnaire with the answers in the appendices)

In examining the responses to our questionnaire, several key insights emerged, shedding light on the transformative impact of the COVID-19 pandemic on dental education. A substantial 75.6% of learners acknowledged their involvement in dentistry faculties or clinics between 2020 and 2022, while an overwhelming 82.9% reported a discernible change in their learning or working experiences during this critical period.

Delving into the changes within dental schools, a nuanced narrative unfolded. The transition towards more online activities and advancements in dental technology was noted, albeit accompanied by a diminishing emphasis on practical classes. The reduction in hands-on experiences, particularly during the initial year with a shift to exclusive online courses, significantly affected the teaching and learning process. The repercussions extended into 2021, where practical activities were gradually reintroduced, albeit with noticeable disruptions. A decrease in patient visits to the university, rigorous infection control measures, and heightened reliance on personal protective equipment were additional facets of this transformative period. While scientific knowledge witnessed an uptick, there was a concurrent decline in the quality of the learning experience, reflecting challenges in maintaining engagement.

The survey also brought to light the substantial shift in educational delivery methods. Notably, 81% of respondents indicated the absence of online classes before the pandemic, with a subsequent 57.1% experiencing an influx of online classes post-pandemic, and 38.1% reporting a period of exclusively online instruction. The need for personal computing resources was underscored, with 83.3% of learners having to procure their own computers, highlighting the challenges associated with remote learning.

As the educational landscape slowly adjusted to a semblance of normalcy in the latter part of 2022 and 2023, respondents pointed to a range of factors influencing their studies or work. These factors included a return to traditional, in-person practices, extended working hours due to compulsory attendance, and the resumption of pre-clinical and clinical practices. Conversely, challenges persisted, with teachers facing readiness issues for online learning and a varied mix of didactic activities carried out electronically, resulting in a complex amalgamation of experiences.

In essence, the responses captured a nuanced journey through the challenges and adaptations within dental education during the pandemic, showcasing both resilience and vulnerability in the face of unprecedented shifts.

### Artificial intelligence in dentistry workshops

During a structured lecture and subsequent workshop, students delved deep into the expansive applications of AI in dentistry. They explored current research trends and practical applications in the clinical realm, ranging from the detection of various conditions and the analysis of dental radiology interpretations to the optimisation of the dental workflow using AI algorithms. The present-day applications of AI in clinical practice were highlighted, emphasising the reality that AI is already a part of contemporary practice while also underscoring the need for future research and regulations.

Learners could gain knowledge about the implementation of AI by dental professionals. The workshop, characterised more by collaborative dialogues than conventional didactics, highlighted the pivotal role of dental students as the future torchbearers of the profession. These emerging professionals faced questions that challenged their analytical skills, sparking debates on ethical issues. Topics included the potential pitfalls of AI-driven diagnostics, challenges associated with treatment predictions and concerns about algorithmic biases. The discussions touched upon the vast potential of AI, from disease detection and treatment planning to its roles in dental imaging and patient management.

**Fig. 2:**
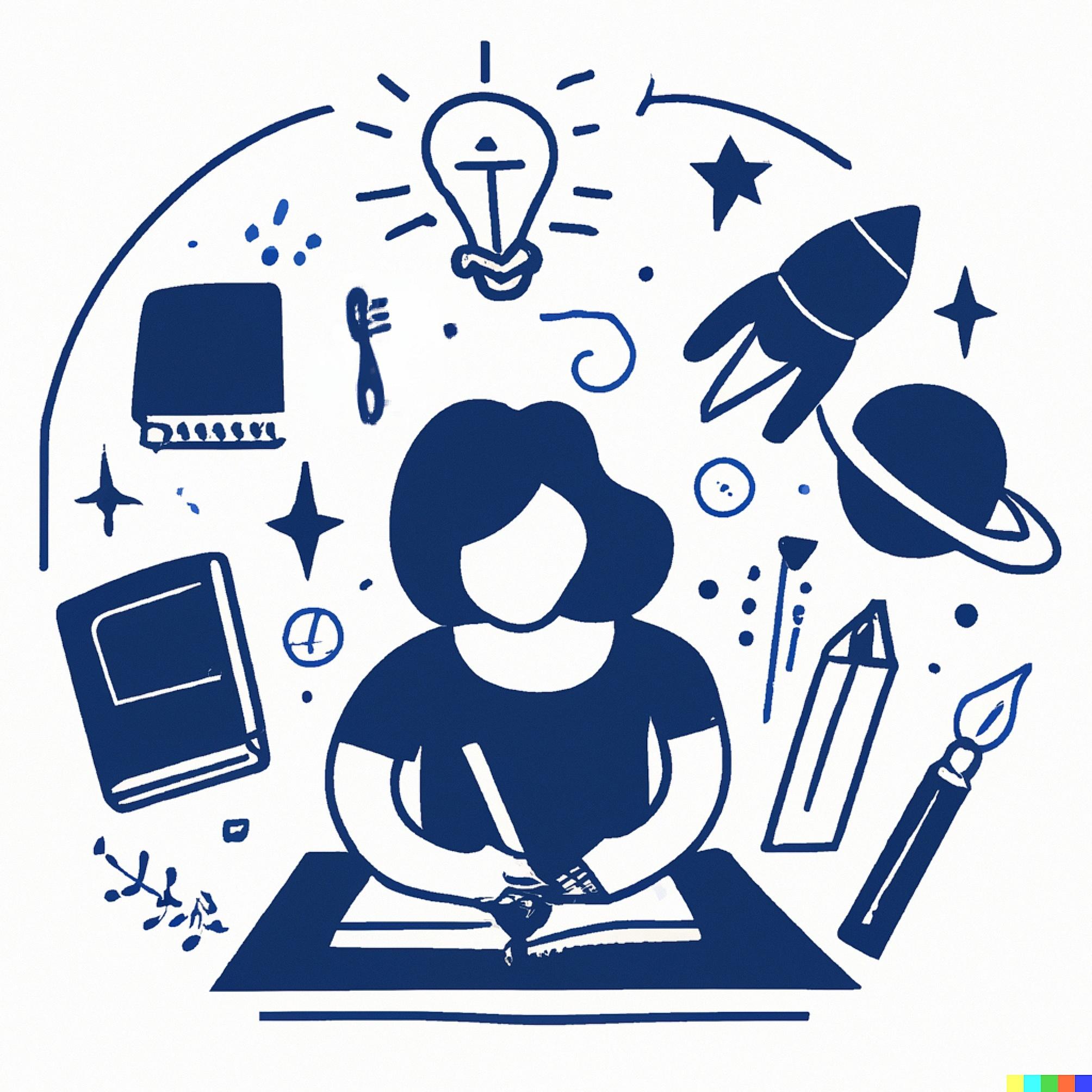
The AI-pixelated feedback of workshops on AI in dentistry via ChatGPT and DALLE2 during the DentalOmics Blended Intensive Programme.

**Fig. 3:**
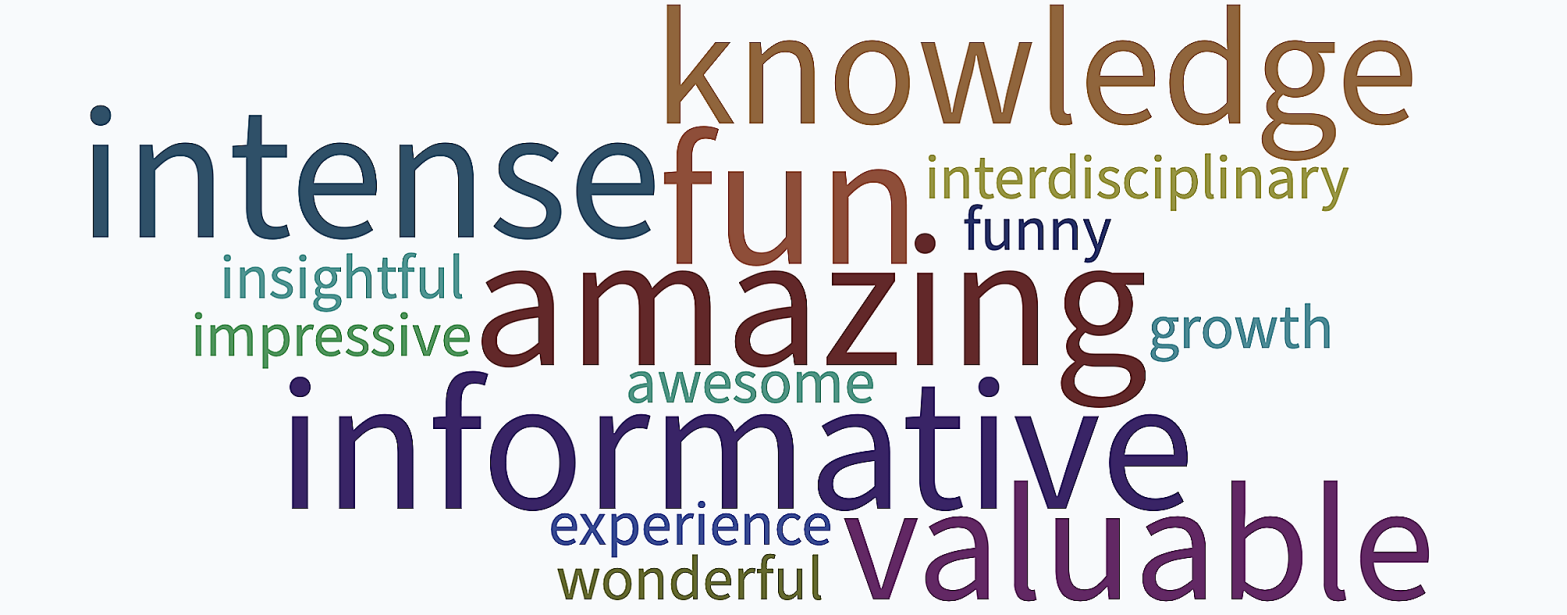
The AI-pixelated feedback of workshops on AI in dentistry via ChatGPT and DALLE2 during the DentalOmics Blended Intensive Programme.

As the workshop concluded, students distilled their week-long experience in Wrocław into individual words, culminating in a word cloud. This collective reflection was then inputted into DALL·E-2, a generative adversarial network model, producing an image that encapsulated their journey through AI in dentistry (Figs. 2 and 3). This exercise highlighted the intersection of AI and academic exploration, emphasising the importance of hands-on experience with such models. The overarching sentiment was clear: for a judicious and effective integration of AI in dentistry, comprehensive engagement with these advanced tools is essential, enabling all stakeholders– students, researchers, faculty and patients– to harness their full potential responsibly.

### Implementation of the tutoring in the dental studies

This part of the training was provided for the dental students attending the tutoring workshop online, followed by one-on-one tutoring meetings on request during the in-person component. Students were asked about their knowledge of tutoring in dentistry and their willingness to collaborate in this field with the teachers. Questions and answers are provided in the appendix.

### Dissemination of the data

Partners were requested to create a description of the BIP on their respective websites, both in the partner country’s native language and, when feasible, in English. The agreed-upon description, formulated in collaboration with partners, was put on the official websites of the participating HEIs. This website was first used for the dissemination of the application process. Following the conclusion of the in-person component at Wroclaw Medical University, HEIs provided short descriptions using the hashtag #DentalOmics and other relevant tags. These descriptions were featured on their institutional websites and official profiles on LinkedIn and/or personal Facebook and/or Instagram profiles of participating students and professors. Additionally, all the partners extended the description of the BIP to their supervisors and incorporated it into their department meetings.

## Discussion

Blended Intensive Programme (BIP) is a relatively new concept of learning introduced by the European Union, involving both online (virtual) components and in-person meetings. The programme is a novel concept. What makes the programme challenging is the inclusion of participants from three different countries where the HEIs provide their credentials for attendance. The complex organisation of both in-person and online components shows the current trend in learning, emphasising the value of transnational idea exchanges as a progressive learning approach. In here presented manuscript, we implemented the transnational learning (education) as per UNESCO/Council of Europe 2001 definition that refers to educational services in which the learners are located in a country different from the one where the awarding institution is based [18]. One of the available summaries of the BIP in medicine was provided by the Iuliu Hațieganu University of Medicine and Pharmacy, Cluj-Napoca, Romania [19]. However, their report focused mostly on the training specifics and electroencephalography topics and not on the teaching methods. In contrast, our manuscript emphasises the methodology and the evaluation of the learning process in dentistry. No publication was found in the online scientific databases presenting a BIP in dentistry; thus, this publication marks the first available evaluation of this learning concept.

Dentistry faculties and dental school students faced the implications of altered learning modalities during the COVID-19 pandemic. The necessity to delay dental procedures to mitigate the risk of salivary bioaerosol spread, thereby reducing the microbiological contamination risk for operators [4], directly impacted the reduction or cancellation of practice hours and, subsequently, students’ clinical exposure. Besides, after the total lockdown, permission to work was restored, and the protection masks and precautions impaired functioning in the dental office. Also, wearing masks led to poorer communication between the dentist, assistant and patients [20]. This phenomenon surely influenced the students similarly as well. Throughout this BIP, students have expressed their dissatisfaction with the limited opportunity to provide patient care during the first year of the pandemic. Our findings align with Schlenz et al. [1], where the authors assert that online education in dentistry cannot replace face-to-face education. The problem with the lack of permission to share the patient data remotely also impairs the possibility of consulting the cases [21, 22]. After the vaccinations were introduced, the dental workers and students were positively adjusted. The vaccination procedures allowed them to return to dental practice, which is mandatory in this profession [23].

However, with limited access to clinical classes, there was a need to establish new learning and educational techniques involving online learning and the utilisation of telemedicine methods to improve the understanding of presented concepts. The authors of this manuscript agree that the condensed curriculum in modern medicine demands effective tools to achieve learning outcomes in a limited timeframe [24]. Blended education has been found, even before the COVID-19 pandemic, to increase the level of education and stimulate effective learning for postgraduate healthcare professionals [25, 26]. Regardless of the limitations, teledentistry is a good model because it allows dental education at a low cost for rural and urban areas and allows some individuals who had no opportunity to broaden their knowledge to self-study [27]. Cooke et al. [17] arrived at similar conclusions about the need for a postgraduate medical approach that accommodates situations where traditional face-to-face teaching is not possible and innovative educational applications are pivotal. Comparable methods were evaluated using blended learning with professional nurses [28]. Although both mentioned manuscripts present results for postgraduate students, there has been relatively less evaluation for undergraduate students. Our work presents a first evaluation of this concept in the field of training undergraduate dental students. Although the investigation of the students’ opinion on teledentistry during the COVID-19 pandemic is very limited, there is still some need for combined online-traditional learning [29].

The term “interprofessional education” has been defined as “occasions when two or more professions learn from and about each other to improve collaboration and the quality of care” [30]. In order to provide this possibility with the support of ERASMUS + KA171 + funding and make the collaboration for professors and students (both learners) more inclusive, an additional group of invited observers was included in the BIP. Lecturers from Rio de Janeiro and Campinas (Brazil) conducted a series of seminars, both online and in-person, in synchronous and asynchronous form. This immersive experience, expanding the academic horizons, was deemed refreshing both by students and professors from the EU region [30, 31]. Moreover, through complete immersion in the transnational professional and student environment, we aimed to promote a sustainable, inclusive network of individuals and organisations [30].

Our programme involved specialists from different professions, leading to the inclusion of interprofessional training in the learning process. As stated by Liaskos et al., a collaborative practice among professions plays a vital role in the patient-centred health services delivery approach [31]. The pandemic itself changed the amount of stress, concerns and behaviours of the teachers as well. The fear of infection interfered with the lecturers, and the will to teach the students has diminished [32]. Furthermore, Liaskos et al. mentioned that an interprofessional team in health has concentrated on two or at most three professions, primarily medicine, nursing and pharmacy [30]. Our BIP provided knowledge from professionals in the field of dentistry, molecular biology, and medicine and soft skills in dentistry.

Overall, based on our direct experience of the Blended Intensive Programme and the experience summarised by the participants and students through their responses to the questionnaire, this type of educational programme received very positive feedback from both sides, the adult and youth components. Furthermore, the possibility of representing these responses through AI opens up the possibility of representing and narrating the experience in a more participatory and inclusive way, promoting a non-verbal and more direct narrative through graphic representation and word cloud. Ultimately, it is conceivable that this type of teaching and training could become an integral part of university systems in the coming years.

Blended Intensive Programmes represent a valuable tool for gathering knowledge and summarising the latest trends in medicine and dentistry. BIPs fill the gap in transnational and interprofessional learning, bringing the possibility of participating in short, well-organised and intense courses where learners can network and receive direct feedback with cutting-edge new learning techniques and a focused approach.

## Conclusions

The COVID-19 pandemic has significantly affected the dental students’ mindset and has necessitated a transformation in teaching methods. The incorporation of tutoring as one-on-one classes can fulfil the students’ desire to collaborate with teachers, especially after an extended period of exclusively online classes and meetings.

Transnational and interdisciplinary approaches are essential between medicine and dentistry in order to ease students into their future roles, instilling an understanding of their significance within the broader context of general medicine, which varies across different countries.

The utilisation of blended learning broadens the spectrum of learning in dental studies. However, in the context of dentistry, a predominantly hands-on discipline, the clinical aspect necessitates in-person delivery to enhance students’ satisfaction with their dental education.

Participation in the BIP contributed positively to thinking about future collaborations among the countries involved and the diseases that affect the oral cavity.

### Electronic supplementary material

Below is the link to the electronic supplementary material.

Supplementary Material 1

## Data Availability

The datasets supporting the conclusions of this article are included in the article and its additional files.
