# Supplementary material for: Blended intensive programme’s implementation in dental education: post-pandemic evolution of learning
Source: BMC Med Educ. 2024 Mar 29;24:352. doi: 10.1186/s12909-024-05301-9 (PMC10981333; doi:10.1186/s12909-024-05301-9)
Supplement: Supplementary file 1 — Supplementary Material 1 [file 12909_2024_5301_MOESM1_ESM.pdf]

Appendix

Here presented are questionnaire answers from the implemented work during the BIP DentalOmics. Learners (teachers and students) were provided with the questionnaire during the online component meant to analyse the effect of the COVID-19 pandemic on the teaching techniques used in the dental studies. The evaluation of the prepared questionnaire employed qualitative and quantitative means using preset questions and a text comment box.

Answers to the questions from the COVID-19 questionnaire were as follows (repetitive answers were excluded):

1. Did you study/work in 2020–2022 in a faculty of dentistry/dental clinic?
   - 75.6% (31) of learners answered "Yes", 19.5% (8) answered "Partly (only one year)"; 4.9% (2) of the learners answered "No"; results are presented in Figure **S1**.

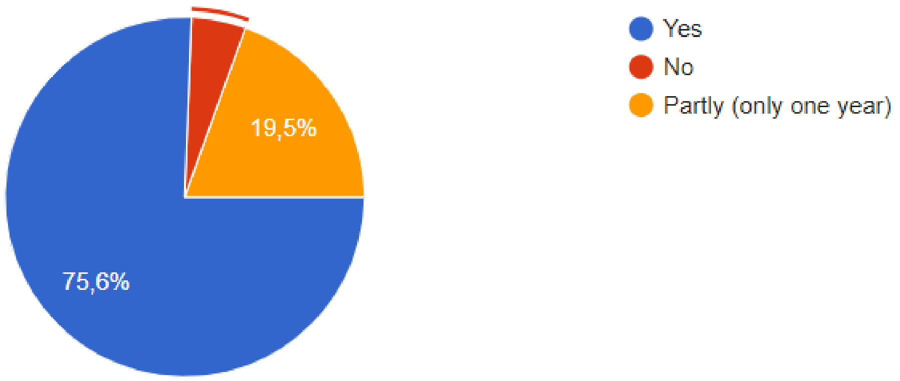

Figure S1. The answers to the question "Did you study/work in 2020-2022 in a faculty of dentistry/dental clinic?" The answers are included in the legend.

2. Did the learning experience/working experience change during this period?
   - 82.9% (34 learners) answered "Yes"
3. What changes did you observe in the dental schools? A total of 28 learners answered the questionnaire in this part, presenting the following outcomes. The ideas of the same kind were implemented in one row, and repetitions of similar ideas were excluded:
   - *More online activities, advancement in dental technology, making the cases smarter;*
   - *Diminished work with patients, classes in person limited to the laboratory practice. The lack of practical classes seriously affected the quality of the teaching and learning process in a negative way. During the first year, the*

teaching activities were reduced to only online courses and practice was interrupted for some months. During 2021, the practical activities were gradually reintroduced. Students in their last year of the dentistry course greatly suffered from this interruption;

- *Fewer patients visited the university due to COVID-19;*
- *More free-time;*
- *There was a list of people entering the university to control the number of people inside the building and the rooms;*
- *More infection control personal equipment, many security measures (we used complete personal protection equipment – PPE);*
- *Unfortunately, the quality of the learning experience decreased. It was less interesting to follow classes, and they were hard to understand;*
- *Increase in scientific knowledge;*
- *Lower influx of people.*

4. Did your school provide online classes before the COVID-19 pandemic?
    - 42 learners answered the question, 81% said “No”, and 11.9% said “Yes”.
5. Did your school provide more online classes after the beginning of the COVID-19 pandemic?
    - 42 learners answered the question, 1% said “No”, 57.1% said “Yes”, and 38.1% said, “For some time, all the classes were provided online”.
6. Did you need to buy your own computer, or was the Dental School helpful in providing you with the required equipment?
    - 83.3% of 42 learners answered, “I had to buy a computer”, and 7.1% [6] answered, “I was able to use a Dental School computer”; results are presented in Figure **S2**.

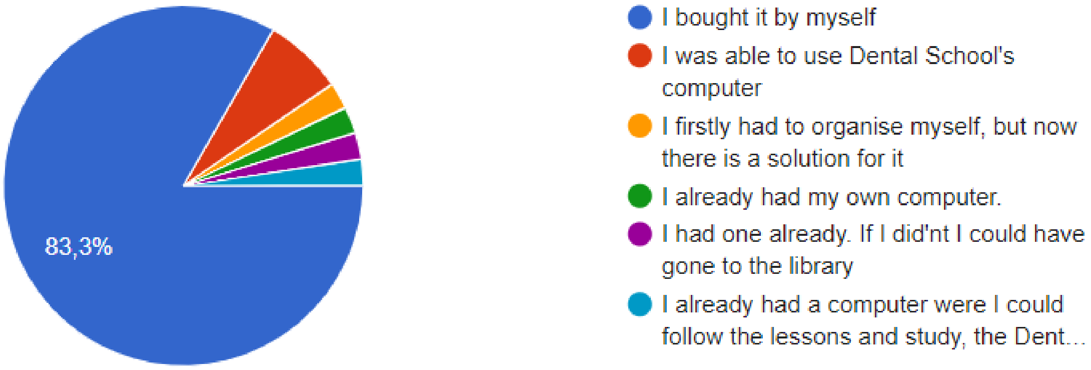

Figure S2. Division of the answers to question "6. Did you need to buy your own computer, or was the Dental School helpful in providing you with the required equipment?" The answers are included in the legend.

7. What is the major factor prevailing in your rate of studies/work in the second part of 2022 and 2023?
   - *No more online studies;*
   - *Very long working days due to compulsory attendance for all lectures and practicals. More interactions during classes with professors and students;*
   - *The feeling of returning to normal, step by step, and having again pre-clinical/clinical practice. We had the opportunity to go back to the normal environment of the university;*
   - *Teachers were not ready for online learning;*
   - *Some didactic activities could be carried out electronically, and time was saved, which was useful for carrying out the clinical part;*
   - *We had more opportunities to attend the wards, and the majority of the professors of the third year (last part of 2022–first part of 2023) were passionate about their field of competence.*

Questions referring to the Tutoring:

1. Have you ever wanted to approach one of your lecturers and ask about possible scientific or tutoring collaboration during your studies? 88.9% of the students answered "Yes", and 11.1% answered "No".
2. If you wanted to approach the lecturer, did you do it? 71.4% answered "No", and 28.6% answered "Yes".

3. If you still wish to approach your tutor, what would be your topic of interest for the collaboration? Students answered “Endodontics”, “To prepare for scientific research and to present it to a conference” and “Dental treatment in people with cancer”.
